# Electroconvulsive Shock Does Not Impair the Reconsolidation of Cued and Contextual Pavlovian Threat Memory

**DOI:** 10.3390/ijms21197072

**Published:** 2020-09-25

**Authors:** Hajira Elahi, Veronica Hong, Jonathan E. Ploski

**Affiliations:** The Department of Neuroscience, The University of Texas at Dallas, 800 West Campbell RD, Richardson, TX 75080, USA; hxe150130@utdallas.edu (H.E.); Veronica.Hong@UTDallas.edu (V.H.)

**Keywords:** reconsolidation, electroconvulsive shock, ECS, ECT, fear conditioning, fear extinction, consolidation

## Abstract

Existing memories, when retrieved under certain circumstances, can undergo modification through the protein synthesis-dependent process of reconsolidation. Disruption of this process can lead to the weakening of a memory trace, an approach which is being examined as a potential treatment for disorders characterized by pathological memories, such as Post-Traumatic Stress Disorder. The success of this approach relies upon the ability to robustly attenuate reconsolidation; however, the available literature brings into question the reliability of the various drugs used to achieve such a blockade. The identification of a drug or intervention that can reliably disrupt reconsolidation without requiring intracranial access for administration would be extremely useful. Electroconvulsive shock (ECS) delivered after memory retrieval has been demonstrated in some studies to disrupt memory reconsolidation; however, there exists a paucity of literature characterizing its effects on Pavlovian fear memory. Considering this, we chose to examine ECS as an inexpensive and facile means to impair reconsolidation in rats. Here we show that electroconvulsive seizure induction, when administered after memory retrieval, (immediately, after 30 min, or after 1 h), does not impair the reconsolidation of cued or contextual Pavlovian fear memories. On the contrary, ECS administration immediately after extinction training may modestly impair the consolidation of fear extinction memory.

## 1. Introduction

Retrieved memories can undergo updating or modification under certain circumstances. Specifically, when an existing memory is retrieved or reactivated, it may become labile or destabilized, necessitating the protein synthesis process of reconsolidation to re-stabilize the memory [1,2,3,4]. The retrieval of a memory can cause the memory engram neurons to undergo another bout of plasticity, which can weaken or strengthen the memory through synapse reorganization [5,6,7,8]. If a protein synthesis inhibitor is administered immediately after memory retrieval, the reconsolidation process can be inhibited, weakening the memory. This phenomenon has been shown to occur across multiple memory types and documented using a variety of species [9,10,11,12,13]. To date, there is a plethora of information available on the subject curated by labs across the globe, and many believe this process has the potential to be harnessed as a treatment for disorders characterized by maladaptive memories, such as anxiety disorders and drug addiction [14,15,16,17].

Current interest in the phenomenon of reconsolidation was kindled by the publication of Karim Nader’s influential work in 2000 [1]. In this study, the administration of a protein synthesis inhibitor immediately after a reminder cue for an auditory fear memory resulted in amnesia. Notably, the reported memory loss was induced outside of the accepted consolidation window, a quality that piqued the attention of memory researchers. This study was followed by replications, which led to the determination of boundary conditions, molecular mechanisms, and attempts at translations into the clinical setting; this is the birth of the reconsolidation field as we know it today.

Notably, however, the first study on reconsolidation was conducted a few decades earlier, in which electroconvulsive shock (ECS) was delivered immediately following a retrieval trial that selectively reactivated an established memory trace [18]. The first documentation of successful memory modification outside of the consolidation window, this study was followed by attempts to reproduce the finding and examine its efficacy in other models of memory. However, a direct replication attempt conducted by another group several months later was unsuccessful in reproducing this critical finding [19]. Since then, other studies examined the use of ECS in disrupting reconsolidation, with mixed success (Table 1). Over time, interest in the phenomenon gradually dissipated, until it was revisited at the turn of the century. At that point, however, researchers opted for pharmacological manipulations to interfere with reconsolidation, and very few studies used ECS.

In the past two decades, protein synthesis inhibitors such as anisomycin and cycloheximide have been frequently used to disrupt reconsolidation, with evidence that both can be delivered intracranially or systemically to block memory restabilization in preclinical models. The beta-adrenergic antagonist propranolol [20], the glucocorticoid antagonist mifepristone [21,22], and the benzodiazepine midazolam [23], have also been shown to interfere with restabilization when administered intracranially and systemically. Unfortunately, recent studies have highlighted that systemic administration of these drugs may not be very effective at disrupting reconsolidation [24,25,26]. Notably, our laboratory has not been successful with impairing the reconsolidation of cued fear memories through systemic administration of these drugs (Supplementary Figure S1 and unpublished observations). Although we have had consistent success with intracranial administration of anisomycin in mice and rats, this procedure requires surgical implantation of cannula, which comes with obvious drawbacks [27]. No doubt, preclinical and clinical research on reconsolidation would benefit greatly from a robust restabilization blocker that could be administered without requiring invasive intracranial access.

One of the most common behavioral paradigms currently used in reconsolidation studies is simple Pavlovian fear/threat conditioning, in which an animal learns to associate a tone and/or context with an aversive foot shock. The strength of the memory is measured on a later test by the amount of freezing behavior exhibited towards the associated cue or stimulus, an inherent response that is typical of rodents exhibiting conditioned fear [28]. While the reconsolidation work using ECS as the amnestic agent has been conducted using various behavioral models, no study examined its utility, or lack thereof, in this exact paradigm. In comparison to pharmacological agents, ECS offers the benefit of being an inexpensive and simpler tool that is relatively easy to administer. Therefore, we sought to determine whether it could be used as a facile substitute for the interventions commonly in use today. Because there was some evidence that ECS could be used to disrupt reconsolidation, and it is well known to induce brain-wide neural plasticity [29], we expected ECS to interfere with the reconsolidation of Pavlovian fear memories. However, here we show that ECS is not effective at attenuating the restabilization of memories modeled by the behavioral paradigms frequently used today.

We also examined the effects of ECS on extinction learning, another mechanism by which memories can be weakened. Rather than altering the original memory, extinction experiences form a new trace that inhibits the original, resulting in attenuated conditioned responses [30,31]. As reconsolidation and extinction are closely related phenomena, and differences in their induction are driven by small variations in stimulus exposure time, we wondered whether our contextual paradigm might be engaging extinction processes. Because ECS has been shown repeatedly to disrupt memory consolidation [32,33,34,35] and extinction is a form of new learning, one might expect ECS to impair fear memory extinction. In fact, such an effect has been described in a study using an appetitive task, showing that ECS interfered with the extinction of a learned bar-press response [36]. We tested whether this effect could translate to the memory model we use here, specifically in the extinction of contextual fear. Although we find that extinction processes are retained following ECS delivery, we show that they may be modestly hindered by this manipulation and result in weakened extinction consolidation.

## 2. Results

### 2.1. ECS Does Not Interfere with the Reconsolidation of Cued Auditory Fear Memory in Male and Female Rats

**Figure 1:**
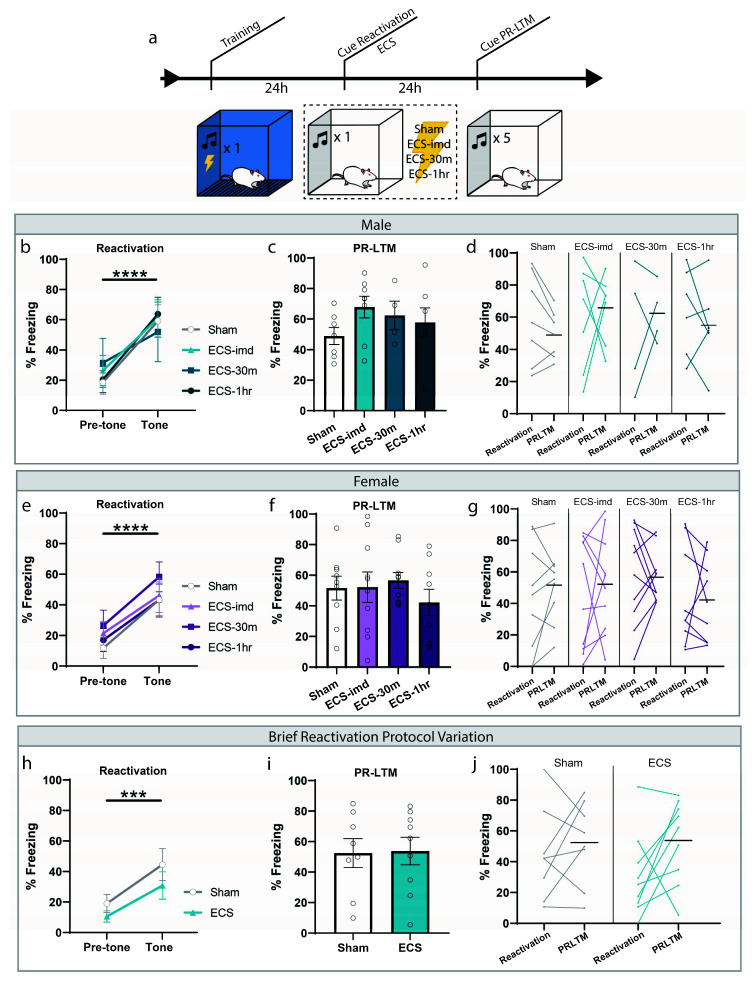
ECS delivery after cued reactivation does not disrupt reconsolidation of an auditory fear memory. (**a**) Schematic outline of experimental procedures used for the cue-ECS experiments. (**b**,**e**) Reactivation graphs indicate that all groups formed an aversive memory associated with the tone. (**c**,**f**) Post-reactivation long-term memory LTM (PR-LTM) graphs show no difference in freezing between the groups resulting from ECS treatment. (**d**,**g**) A within-subjects comparison showed no change in any group across days. (**h**–**j**) Modifying the duration of the reactivating tone and session prior to ECS does not result in amnesia either. *** *p* < 0.001, **** *p* < 0.0001, error bars = standard error of the mean (SEM). Horizontal bar = PR-LTM mean. “Pre-tone” is defined as the acclimation period during reactivation, prior to the tone presentation.

We initially sought to determine whether ECS could impair the reconsolidation of a typical auditory fear memory, and what the window of interference, if one exists, looks like. Based on the available literature, we expected to see an amnestic effect when a seizure is induced immediately following memory reactivation. Rats were trained and tested in accordance with the fear conditioning protocol commonly used to study reconsolidation mechanisms today (Figure 1a). Training consisted of a single presentation of a 30-s tone (CS) co-terminating with a mild foot shock (1 s, 0.75 mA). These parameters were chosen to form a memory weak enough to be generally susceptible to the effects of reconsolidation disruptors. Twenty-four hours later, the memory was reactivated using a retrieval session, in which rats were placed in a novel context and presented with a reminder CS (Figure 1b,e). All groups successfully learned the association, indicated by the significant increase in freezing to the tone (repeated-measures ANOVA, Male: F(1,20) = 48.35, *p* < 0.0001; Female: F(1,34) = 48.46, *p* < 0.0001), and there was no difference between groups for freezing (one-way ANOVA, Male: F(3,20) = 0.12, *p* = 0.9478; Female: F(3,34) = 0.45, *p* = 0.7162). After the reactivation trial, rats were separated into four treatment groups: ECS delivered immediately (ECS-imd), 30 min (ECS-30m) or 1 h (ECS-1h) after the session, or sham stimulation. Only animals exhibiting a full tonic-clonic seizure induced by ECS were included in the analysis. The following day, the animals were returned to the testing context for a post-reactivation long-term memory (PR-LTM) test to determine the effects of ECS on memory persistence (Figure 1c,f). On the PR-LTM test, there was surprisingly no significant difference between any of the groups, whether the experiment was conducted using male or female rats (repeated-measures ANOVA, Male: F(3,20) = 0.90, *p* = 0.4578; Female: F(3,34) = 0.74, *p* = 0.5359). As there appeared to be some variability between the groups at reactivation, we conducted a within-subjects analysis to determine freezing changes for each group across the testing days (Figure 1d,g). However, no difference was observed in any of the groups (repeated-measures ANOVA, Male: F(3,20) = 0.58, *p* = 0.6340; Female: F (3,34) = 0.23, *p* = 0.8730).

We were surprised by this result, as we were expecting to see a reduction in freezing resulting from ECS treatment, since this effect was shown in several studies. We thought that our reactivation procedure may not be optimally designed to allow for such an interference and conducted an additional experiment that mimicked the conditions used by Misanin et al. [18] more closely. Instead of presenting a full-length 30 s CS, we shortened the duration to several seconds such that the tone was only played briefly, with the ECS delivered immediately after tone termination (see methods). Using these conditions, which shortened the time between CS onset and ECS delivery, we were still unsuccessful in inducing amnesia, as there was no difference between the ECS and sham groups at PRLTM (repeated-measures ANOVA, F(1,15) = 0.01, *p* = 0.9212), nor was there a within-subjects change (repeated-measures ANOVA, F(1,15) = 0.89, *p* = 0.3609) (Figure 1h–j). Although much of the literature establishes the prediction that ECS delivered immediately after reactivation should interfere with the reconsolidation of an auditory fear memory, these results show that ECS is not effective at inducing amnesia for such a memory, contrary to our expectations.

### 2.2. ECS Does Not Interfere with the Reconsolidation of Context Fear Memory in Male and Female Rats

**Figure 2:**
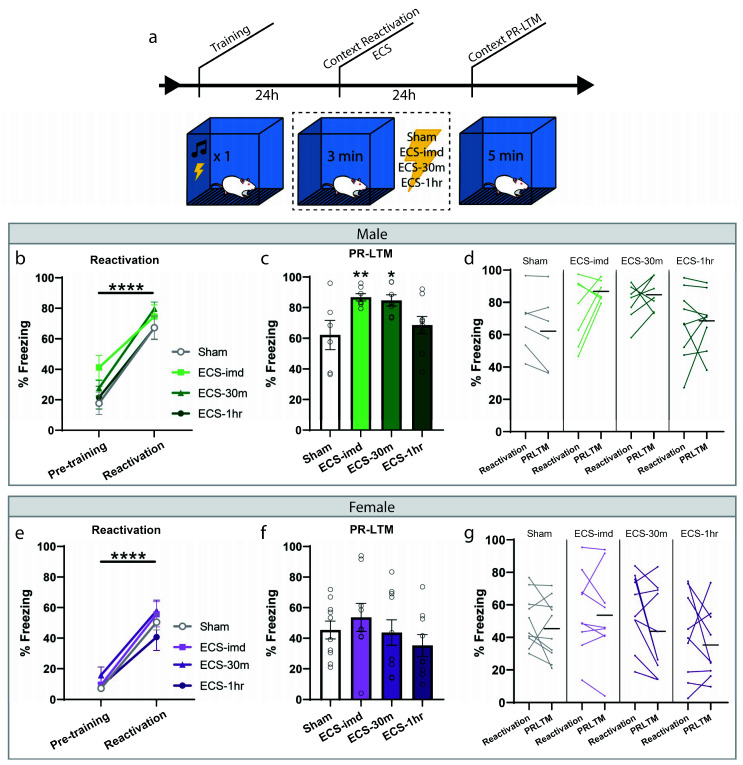
ECS delivery after contextual reactivation does not disrupt reconsolidation of a contextual fear memory. (**a**) Schematic outline of experimental procedures for the context-ECS experiments. All animals acquired an aversive contextual memory, as measured at reactivation (**b**,**e**). PR-LTM freezing data for the male (**c**) and female (**f**) cohorts show no amnesia resulting from ECS. The ECS-imd and ECS-30m groups from the male cohort display significantly greater freezing than the sham group (**c**). A within-subjects comparison showed no change in any group across days (**d**,**g**). * *p* < 0.05, ** *p* < 0.01, **** *p* < 0.0001, error bars = SEM. Horizontal bar = PR-LTM mean. “Pre-training” is defined as the context acclimation period during training, prior to delivery of the tone-shock pairing.

We then wondered whether ECS could be used as an intervention for contextual fear memories, if not for cued. For this experiment, fear conditioning was done as before; however, the reactivation session consisted of a short re-exposure to the training context (Figure 2a). All animals acquired sufficient learning, indicated by the significant increase in freezing to the context during the reactivation trial (repeated-measures ANOVA, Male: F(1,25) = 92.39, *p* < 0.0001; Female: F(1,34) = 140.26, *p* < 0.0001) with no difference between groups (one-way ANOVA, Male: F(3,25) = 0.78, *p* = 0.5214; Female: F(3,34) = 1.09, *p* = 0.3687). The animals were then separated into the same conditions as before, ECS-imd, ECS-30m, ECS-1h and sham, and were tested for memory strength with a PR-LTM context test (Figure 2c,f). Again, we observed no deleterious effect of ECS on reconsolidation, bolstering our conclusion on its nonutility. However, in this instance, there was a significant group effect at PR-LTM in the data gathered from the male cohort (repeated-measures ANOVA, Male: F(3,25) = 4.30, *p* = 0.0141; Female: F(3,34) = 0.91, *p* = 0.4441), where, surprisingly, the immediate and 30 min ECS groups froze significantly more than the sham group (post hoc Fisher’s Least Significant Difference LSD, sham vs. ECS-imd *p* = 0.0075; sham vs. ECS-30m *p* = 0.0132; sham vs. ECS-1h *p* = 0.4293). We wondered whether this difference was due to memory strengthening by means of reconsolidation updating. However, a within-subjects analysis showed no change within any group across testing days (Figure 2d,g) (repeated-measures ANOVA, Male: F(3,25) = 1.36, *p* = 0.2771; Female: F(3,34) = 0.72, *p* = 0.5495), indicating that the difference seen at PR-LTM is not due to memory strengthening and may be explained by the inherent variability in learning between groups. 

**Figure 3:**
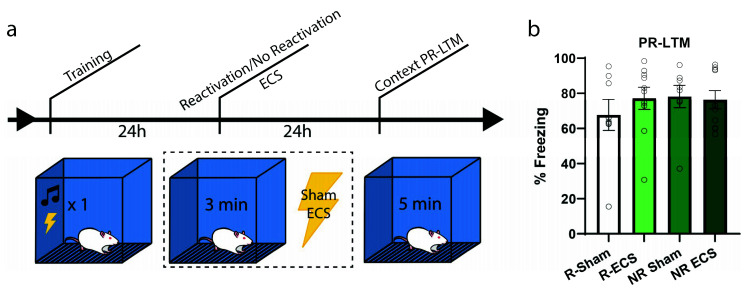
ECS causes no reactivation-dependent deficits of a contextual fear memory. (**a**) After training, two groups were subjected to a reactivation session followed by ECS or sham stimulation, while the remaining two groups received stimulation without memory reactivation. (**b**) There was no difference in freezing between the groups at PR-LTM (**b**). Error bars = SEM.

To further examine whether there might be an increase in freezing due to ECS and if the effect is dependent upon memory reactivation, our next experiment followed the same context training and testing procedures; however, only half of the animals received a reactivation trial (Figure 3a). Each group was further divided such that half of the animals were given ECS while the others sham, resulting in the following groups: Reactivation-Sham (R-Sham), Reactivation-ECS (R-ECS), No Reactivation-Sham (NR-Sham), and No Reactivation-ECS (NR-ECS). As we expected by now, there was no freezing deficit at PR-LTM resulting from ECS (two-way ANOVA, main effect of “ECS” F(1,32) = 0.32, *p* = 0.5733), and all treatment groups behaved similarly regardless of memory reactivation (main effect of “reactivation” F(1,32) = 0.52, *p* = 0.4742). The variability observed between this result and that of the previous context experiment can be explained by differences in reactivation freezing, and further indicate that ECS after reactivation does not drive changes in memory strength in any direction; the memories are neither weakened nor strengthened.

### 2.3. ECS May Modestly Interfere with the Consolidation of Context Fear Extinction Memory 

**Figure 4:**
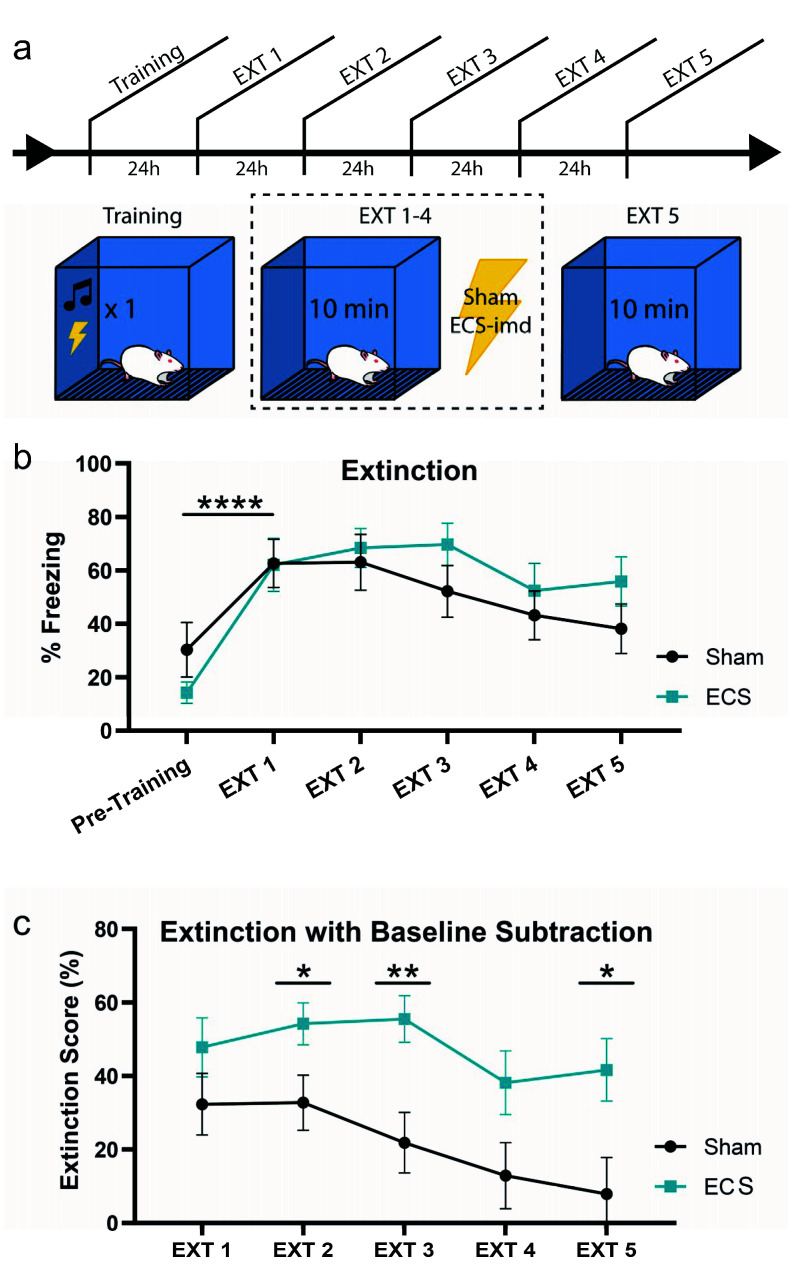
ECS may modestly impair extinction learning. (**a**) Schematic outline of experimental procedure testing the effects of ECS on extinction learning. (**b**) While both groups extinguish the fear memory, the sham group may extinguish the fear faster than the ECS group. This is indicated by a reduction in statistically significant differences between pre-training (baseline) and extinction session freezing. (**c**) Extinction data from (**b**) with baseline subtraction (extinction score) for the groups on each day indicate that the ECS group continues to show high levels of freezing across days, while the sham group displays a steady decrease to baseline freezing levels, such that the difference in fear extinction between the groups is significant at EXT 2, 3, and 5. * *p* < 0.05, ** *p* < 0.01, **** *p* < 0.0001, error bars = SEM. “Pre-training” is defined as the context acclimation period during training, prior to delivery of the tone-shock pairing.

Next, we turned our attention to the possibility that ECS may be disrupting the consolidation of extinction learning, as it is possible that the unreinforced retrieval of a fear memory would drive extinction learning. To test this possibility, we trained animals as before and delivered ECS or sham immediately after context extinction trials for four days (EXT 1–4), giving a final memory test on the fifth day (EXT 5) (Figure 4a). All animals acquired the memory, as indicated by the increase in freezing at EXT1 from pre-training levels (repeated-measures ANOVA, F(1,14) = 45.76, *p* < 0.0001) (Figure 4b). An analysis conducted across all extinction days did not show any difference between the groups (repeated-measures ANOVA F(1,14) = 0.65, *p* = 0.4327), and that extinction occurred across all subjects (F(4,56) = 7.55, *p* < 0.001).

Interestingly, however, pairwise comparisons of the freezing levels for each group between days showed that while the sham group reached pre-training freezing levels by EXT 3, the ECS group was still freezing at a significantly higher rate even at EXT 5. To visualize these differences more clearly, we plotted the data for each day as a difference from baseline, or pre-training, freezing levels (Figure 4c). This was done by calculating an “extinction score” for the groups on each extinction day, such that a low score would indicate greater extinction, while a higher score would indicate the opposite (see methods). As shown in the previous graph, the sham group returns to baseline freezing levels by EXT 5, while the ECS group still exhibits high levels of fear to the context. Pairwise comparisons of the extinction scores for each day show that the difference in extinction between the two groups is significant at EXT 2, 3, and 5 (EXT2 t_(1,14)_ = 2.17, *p* = 0.0480; EXT3 t_(1,14)_ = 3.08, *p* = 0.0081; EXT5 t_(1,14)_ = 2.48, *p* = 0.0265). Taken together, these data indicate that while ECS does not result in a full blockade of extinction, it may modestly impair the rate at which the conditioned responses to the associated context decrease to match baseline freezing levels.

## 3. Discussion

Here we demonstrate that the administration of ECS immediately, 30 min and 1 h after the retrieval of a context or cue fear memory does not impair the reconsolidation of the conditioned fear. We approached the design in a variety of ways using both male and female subjects, but the memories remained intact across all experiments. Surprisingly, we observed an inflation of memory strength resulting from the immediate administration of ECS after contextual reactivation when measured at PR-LTM, (i.e., increase in freezing) (Figure 2). We initially hypothesized that this inflation may be due to ECS strengthening the retrieved memory via reconsolidation updating. However, with further analysis and experimentation, we found that the freezing levels across days were not consistent with reconsolidation-driven memory updating (Figure 2d, Figure 3). Alternatively, we thought that ECS might impair the consolidation of extinction memory instead, since the average fold differences in the PR-LTM data indicated such a possibility. One would even expect ECS to interfere with extinction learning as it would be consistent with the well-known phenomenon of ECS inhibiting memory consolidation [32,43], and in agreement with a study showing an ECS-induced deficit in the extinction of an appetitive task [36]. As the 3 min exposure time during the context reactivation session was likely not long enough to consistently engage extinction processes, we conducted a separate experiment to examine this effect specifically. To test this, we administered ECS after several 10 min extinction trials for a context fear memory (Figure 4). While both groups appeared to extinguish the fear memory, our extinction score analysis showed that ECS delivery significantly impaired the rate of extinction. Whereas the sham group had returned to baseline freezing levels by day 3, the ECS group still exhibited significantly high freezing at day 5. This supports our conclusion that ECS may modestly interfere with the extinction rather than interfere with the reconsolidation of Pavlovian fear memories. More research will be needed to determine the extent of ECS-induced impairment on fear memory extinction.

The early studies that described ECS as an effective tool to attenuate reconsolidation were done using different behavioral paradigms than the ones we have tested here, which are commonly used in fear experiments conducted today. While the first of these did study associative conditioned fear, the strength of the fear memory was determined by performance on an appetitive task [18]. Water restricted animals were trained with a tone-shock pairing, and the tendency to drink water from a spout in the testing chamber was used to measure fear associated with the CS. The two other studies conducted by the same group examined cue-dependent amnesia in a spatial memory task [38,39]. The experimenters trained animals on a K maze and tested the contingency of ECS-induced amnesia on salient cues associated with the task, such as handling and placement in the maze start chamber. Notably, these studies delivered the ECS to subjects inside the testing boxes immediately after CS presentation, which is different from how reconsolidation interventions are typically administered. Although we predicted that we would achieve an amnestic effect using our experiment design similar to that described in these studies, our data show that ECS does not functionally interfere with reconsolidation for cued and contextual Pavlovian fear memory when memory strength is determined by freezing behavior.

The tonic-clonic seizure induced by ECS results in brain-wide neural activation that can induce large-scale synaptic modification. Consistent with this, a single administration of ECS has been shown to induce gene expression and morphological changes in neurons across brain regions, including the hippocampus and frontal cortex [29,44,45,46,47,48,49]—two areas critical for fear learning and memory modification via reconsolidation and extinction [28,50]. It is not surprising that ECS can disrupt memory processes; however, the exact mechanism by which this occurs is unclear. Given that an unconsolidated memory trace is in its most vulnerable state, the non-specific changes in synaptic strength and gene expression driven by ECS administration likely disrupt the processes involved with memory stabilization [32,33,34,35,51]. While reconsolidation and consolidation certainly share many characteristics, reconsolidation is not an exact recapitulation of consolidation [52], and likely does not possess the same degree of vulnerability as an unconsolidated memory trace. Our data showing that ECS administration did not impair reconsolidation of context and cued fear memories are consistent with this.

Our findings should not cast doubt on the existence of reconsolidation in general. Rather, they showcase that disruption of this phenomenon can only be achieved by certain manipulations delivered in a very specific manner. We highlight the need to identify effective drugs or interventions that can be used to target the reconsolidation process in a way that is easily translatable to the clinical setting. Accumulating evidence supports the hypothesis that the restabilization phase of reconsolidation is ultimately blocked by the interference of α-amino-3-hydroxy-5-methyl-4-isoxazolepropionic acid receptor AMPA receptor reinsertion into the post-synaptic membrane [53]. As existing reconsolidation disruptors function by affecting the process indirectly, such as through protein synthesis inhibition, it would be useful to identify agents that impair this process directly.

## 4. Materials and Methods

### 4.1. Subjects

Sprague Dawley rats weighing between 175 and 250 g (Taconic) were individually housed and maintained on a 12 h light/dark cycle, with food and water provided ad libitum throughout the experiment. Experiments were conducted during the light hours of the cycle, between 6:00 a.m. and 6:00 p.m. For all experiments, groups were balanced for age and weight. Animal use procedures were in accordance with the National Institutes of Health Guide for the Care and Use of Laboratory Animals and were approved by the University of Texas at Dallas Animal Care and Use Committee IACUC # 10-04, 17 April 2020. The initial cue and context reconsolidation experiments (Figure 1b–g and Figure 2) were conducted with a separate cohort for each sex. After determining that there were no sex-specific effects of ECS, the remaining experiments were done using male subjects only.

### 4.2. Behavior

Coulbourn Instruments fear conditioning system was used in conjunction with Freezeframe 4 software (Actimetrics, Wilmette, IL, USA) to deliver tones and shocks and perform unbiased behavioral analysis. Freezing behavior was used as a measure of fear memory strength and was defined as the complete absence of all movement except that required for respiration. At the start of each day, animal cages were transferred from the vivarium facility to a holding room in our lab, and the subjects were given a 30 min acclimation period before removal for experimentation. Fear conditioning and testing were done in four identical sound attenuating chambers, in a room adjacent to the holding room. For three days prior to training, animals were habituated to the experimenters, conditioning chambers, and the training context.

- Training: Animals were trained in a distinct contextual environment (training context) characterized by blue walls, bright lighting, and a grid floor, cleaned with 70% isopropanol before each round. After a 3 min acclimation period (pre-training), animals received a single conditioning trial that consisted of a 30 s, 5 kHz, 75 dB tone co-terminating with a 1 s, 0.75 mA foot shock. The animals remained in the conditioning context for another minute before being returned to their home cages.
- Context memory reactivation: Memory was reactivated by reintroducing the animals to the conditioning context for 3 min.
- Cued memory reactivation: Memory was reactivated by placing the animals in a novel environment characterized by clear walls, red lighting, textured floor, and a vanilla scent (testing context). For experiments using the typical reactivation conditions, after a 2 min acclimation period (pre-tone), animals were given one presentation of the conditioned stimulus (30 s, 5 kHz, 75 dB tone), and removed from the chamber after 30 s. For the experiment using a shorter reactivation session, animals were given a 10 s presentation of the tone and were removed from the chamber immediately.
- PR-LTM Test: For contextual memory, animals were returned to the training context for 5 min, and memory strength was determined by the amount of time spent engaged in freezing behavior. For cued memory, animals were placed in the testing context and were given 5 equally interspaced CS presentations delivered after a 150 s acclimation period. The percentage of time spent freezing during these five tones was averaged.
- Context extinction: Extinction trials consisted of 10 min exposures to the context, and freezing was averaged for the duration of the trial. To determine the changes in freezing from baseline levels, an “extinction score” was calculated by subtracting pre-training freezing from each day of extinction (ExtX—pre-training).
- Statistical analyses: One-way ANOVA, repeated-measures ANOVA, and unpaired *t*-tests were used to calculate the statistical significance between groups where appropriate. Post hoc analyses were carried out using uncorrected Fisher’s Least Significant Difference LSD.

### 4.3. Electroconvulsive Shock (ECS)

ECS was delivered to subjects as previously described [54,55]. Specifically, ECS was administered through ear-clip electrodes with a pulse generator (ECT Unit 57800-001; Ugo Basile, Comerio, Italy) using the following parameters: frequency, 120–130 Hz; pulse width, 0.5 ms; shock duration, 2.0–2.9 s; current 99 mA. Sham animals were treated identically to ECS animals but were not delivered any shock. ECS was delivered in the testing room where fear conditioning occurred, except for the contextual experiment testing retrieval dependence, in which ECS was delivered in another room. Only animals exhibiting tonic-clonic seizures were included in analyses. The number of animals excluded for displaying “clonic-only” seizures is as follows, delineated by the respective figure for each experiment: Figure 1c—two; Figure 1f—one; Figure 2c—four. Our results and conclusions are not affected by the inclusion of these animals (Supplementary Table S1). For the extinction experiment, two animals did not display complete seizures on one day of the experiment; because they exhibited tonic-clonic seizures on all other days, they were not excluded from analysis.

## Figures and Tables

**Table 1:** Studies that have used electroconvulsive shock (ECS) to interfere with reconsolidation.

Year	Subject	Learning Model	Amnesia?	Reference
1968	Rat	Auditory fear conditioning, memory tested using appetitive (drinking) task	Yes	[18]
1969	Rat	Auditory fear conditioning, memory tested using appetitive (drinking) task	No	[19]
1972	Rat	Auditory fear conditioning, memory tested using appetitive (drinking) task	Yes	[37]
1972	Rat	K maze (spatial memory)	Yes	[38]
1973	Rat	K maze (spatial memory)	Yes	[39]
1976	Depressed patients	Item recognition memory, remote memory recall	No	[40]
2013	Mice	Cocaine/Food CPP, Passive Avoidance	Yes	[41]
2014	Depressed patients	Recall of emotionally aversive slide-show stories	Yes	[42]

## Supplementary Materials

The following are available online at https://www.mdpi.com/1422-0067/21/19/7072/s1, Table S1. Data Analysis of Experiments with the Inclusion of Animals Exhibiting “Clonic-only” Seizures; Figure S1. Midazolam does not disrupt reconsolidation of an auditory fear memory and associated methods.

## Abbreviations

ECS	Electroconvulsive shock
ECT	Electroconvulsive therapy
SEM	Standard error of the mean
CS	Conditioned Stimulus
