# Supplementary material for: Electroconvulsive Shock Does Not Impair the Reconsolidation of Cued and Contextual Pavlovian Threat Memory
Source: Int J Mol Sci. 2020 Sep 25;21(19):7072. doi: 10.3390/ijms21197072 (PMC7582782; doi:10.3390/ijms21197072)
Supplement: Supplementary file 1 [file ijms-21-07072-s001.pdf]

Supplementary Materials

Table S1. Data Analysis of Experiments with the Inclusion of Animals Exhibiting "Clonic-only" Seizures

Experiment	RM ANOVA of PR-LTM
Cue Recon; Male group (figure 1c)	F(3,22) = 1.15, p=0.3505
Cue Recon; Female group (figure 1f)	F(3,35) = 0.64, p=0.5929
Context Recon; Male group (figure 2c)	F(3,29) = 2.86, p=0.0542

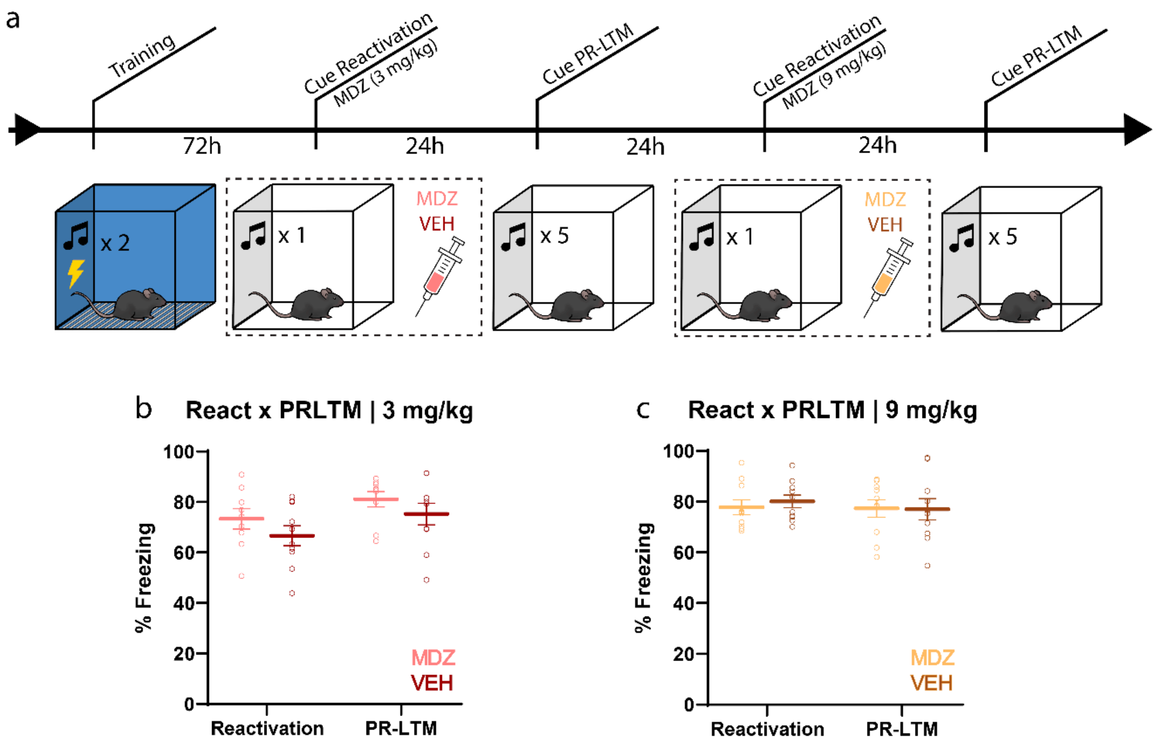

Figure S1. Midazolam does not disrupt reconsolidation of an auditory fear memory. (a) Schematic outline of experimental procedures used. MDZ was administered at 3 mg/kg (b) and 9 mg/kg (c) doses immediately after memory reactivation. Animal group assignments remained the same for the duration of the experiment. Neither doses of MDZ resulted in amnesia for the cued memory when tested at PR-LTM (repeated-measures ANOVA, 3 mg/kg: F(1,17)=1.174, *p=.2937*; 9 mg/kg: F(1,18)=0.004, *p=0.9515*). Error bars = SEM.

Supplementary Methods (Midazolam Reconsolidation Experiment)

Subjects

Three-month-old male C57BL/6 mice (Jackson Laboratory) were individually housed and maintained as described in the main text.

Behavior

Fear conditioning was conducted as described in the main text, with the following differences:

Animals were habituated for two days prior to training to the experimenter, conditioning chambers and both training and testing contexts.

Training: Animals were trained in a distinct contextual environment characterized by blue walls, bright lighting, and a grid floor, cleaned with 70% isopropanol before each round. After a 3-minute acclimation period (Pre-Training), animals received two conditioning trials consisting of a 30 s, 5 kHz, 75 dB tone co-terminating with a 2 s, 0.75 mA foot shock. The animals remained in the conditioning context for another minute before being returned to their home cages.

Reactivation: Memory was reactivated by placing the animals in a novel environment characterized by white walls, bright lighting, and a peppermint scent (testing context). Following a 120-second acclimation period, animals were given one presentation of the conditioned stimulus (30 s, 5 kHz, 75 dB tone), and removed from the chamber after 30 s.

PR-LTM Test: Animals were returned to the testing context described above and given 5 equally spaced CS presentations.

Drug Administration

Midazolam Maleate Salt (Sigma-Aldrich) was dissolved in sterile saline (1 mg/mL) and administered intraperitoneally at 3 mg/kg and 9 mg/kg doses immediately after memory reactivation.
